# Roles of hepatic atypical protein kinase C hyperactivity and hyperinsulinemia in insulin‐resistant forms of obesity and type 2 diabetes mellitus

**DOI:** 10.1002/mco2.54

**Published:** 2021-02-25

**Authors:** Mini P. Sajan, Barbara C. Hansen, Mildred Acevedo‐Duncan, Mark S. Kindy, Denise R. Cooper, Robert V. Farese

**Affiliations:** ^1^ Department of Internal Medicine University of South Florida College of Medicine Tampa Florida USA; ^2^ Research Service James A Haley Veterans Administration Medical Center Tampa Florida USA; ^3^ Department of Chemistry College of Arts and Sciences University of South Florida Tampa Florida USA; ^4^ Department of Pharmaceutical Sciences College of Pharmacy University of South Florida Tampa Florida USA; ^5^ Department of Molecular Medicine University of South Florida Tampa Florida USA

**Keywords:** Alzheimer's disease, atypical protein kinase C, BACE1, diabetes mellitus, hyperinsulinemia, insulin, obesity

## Abstract

Diet‐induced obesity, the metabolic syndrome, type 2 diabetes (DIO/MetS/T2DM), and their adverse sequelae have reached pandemic levels. In mice, DIO/MetS/T2DM initiation involves diet‐dependent increases in lipids that activate hepatic atypical PKC (aPKC) and thereby increase lipogenic enzymes and proinflammatory cytokines. These or other hepatic aberrations, via adverse liver‐to‐muscle cross talk, rapidly impair postreceptor insulin signaling to glucose transport in muscle. The ensuing hyperinsulinemia further activates hepatic aPKC, which first blocks the ability of Akt to suppress gluconeogenic enzyme expression, and later impairs Akt activation, further increasing hepatic glucose production. Recent findings suggest that hepatic aPKC also increases a proteolytic enzyme that degrades insulin receptors. Fortunately, all hepatic aberrations and muscle impairments are prevented/reversed by inhibition or deficiency of hepatic aPKC. But, in the absence of treatment, hyperinsulinemia induces adverse events, some by using “spare receptors” to bypass receptor defects. Thus, in brain, hyperinsulinemia increases Aβ‐plaque precursors and Alzheimer risk; in kidney, hyperinsulinemia activates the renin–angiotensin–adrenal axis, thus increasing vasoconstriction, sodium retention, and cardiovascular risk; and in liver, hyperinsulinemia increases lipogenesis, obesity, hepatosteatosis, hyperlipidemia, and cardiovascular risk. In summary, increases in hepatic aPKC are critically required for development of DIO/MetS/T2DM and its adverse sequelae, and therapeutic approaches that limit hepatic aPKC may be particularly effective.

## Graphical abstract

PKC‐ι in primates and its homologue, PKC‐λ in mice, are the major 70‐kDa aPKCs in liver, skeletal muscle, cardiac muscle, and brain and are activated by IRS‐1/PI3K in skeletal and cardiac muscle, but by IRS‐2/PI3K in liver (brain data on IRS are lacking). In muscle and liver, Akt is mainly activated by IRS‐1/PI3K, but, in heart, Akt is mainly and constitutively activated by IRS‐2/PI3K. In liver, Akt selectively phosphorylates FoxO1 and PGC‐1α on the WD40/ProF platform, shown by the shaded area, and, whereas both Akt and aPKC are used for insulin‐stimulated lipogenesis, Akt alone mediates insulin suppression of gluconeogenesis. Indeed, aPKC excess impairs the effect of Akt on FoxO1, PGC‐1a, and gluconeogenesis by displacing Akt from the WD40/ProF platform.

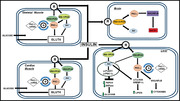

**Abbreviations**
A2‐R: angiotensin‐2 receptor
ACC: acetyl‐CoA carboxykinase
ACPD: 2‐acetyl‐cyclopentane‐1,3,‐diketone
AD: Alzheimer's disease
AMPK: AMP‐activated kinase
aPKC: atypical protein kinase C
Aβ: amyloid‐beta
BACE1: β‐amyloid precursor protein‐cleaving enzyme‐1
DAG: diacylglycerol
DIO: diet‐induce obesity
FA: fatty acids
FAS: fatty acid synthase
FoxO1: forkhead homeobox class‐O1 protein
G6Pase: glucose‐6‐phosphatase
GSK3β: glycogen synthase kinase‐3β
HFD: high‐fat diet
HFF: high‐fat‐fed
ICAPP: H‐imidazole‐4‐carboxamide,5‐amino]‐[2,3‐dihydroxy‐4‐[(phosphono‐oxy)methyl]‐cyclopentane‐[1R‐(1a,2b,3b,4a)]
IGF‐1: insulin‐like growth factor‐1
IL‐1β: interleukin‐1β
IR: insulin receptor
IRS‐1: insulin receptor substrate‐1
IRS‐2: insulin receptor substrate‐2
IRsol: soluble fragment of the insulin receptor
KI: kinase‐inactive
KO: knockout
MetS: metabolic syndrome
mTOR: mammalian target of rapamycin
NFκB: nuclear factor κ‐B
PEPCK: phosphoenolpyruvate carboxykinase
PGC‐1α: peroxisome proliferator‐activated receptor‐gamma (PPARχ) coactivator‐1α
PI: phosphatidylinositol
PI3K: phosphatidylinositol 3‐Kinase
PIP_3_
: phosphatidylinositol‐3,4,5‐(PO_4_)_3_
PKC: protein kinase C
PKC‐λ/ι: protein kinase C‐lambda/iota
PKMζ: protein kinase M‐zeta
PM: plasma membrane
SREBP‐1c: sterol receptor element‐binding protein‐1c
T2DM: type 2 diabetes mellitus
TAG: triacylglycerol
TB/HetλKO: total‐body heterozygous PKC‐λ knockout
TGN: trans‐Golgi apparatus
TNF‐α: tumor necrosis factor‐α
WD40/ProF: WD40/Propeller‐FYVE (WD40/ProF), a 40 kDa protein that contains seven tryptophan (W) ‐ amino‐acid‐amino‐acid‐aspartate (d)‐repeat proteins and one FYVE domain protein (domain in Fab1p, YOTB, Vac1p, and EEA19 early endosome antigen‐1)
βAPP: beta‐amyloid precursor protein
β‐APP: precluding production of Aβ‐peptides

## 1 GENERAL BACKGROUND INFORMATION

To better understand clinical insulin resistance, we will first review how insulin uses signaling factors to control metabolic processes. Initially, insulin binds to the α‐subunit of cell surface insulin receptors (IRs) and conformationally increases the tyrosine kinase activity of transmembraneous β‐subunit, which phosphorylates intracellular tyrosine (Y) residues of adjacent β‐subunits and non‐IR proteins,^1^ most notably, IR substrate‐1 (IRS‐1) and IRS‐2. Thusly, pYXXM motifs are generated in IRS‐1/2 and interact with SH domains of p110 subunits of phosphatidylinositol (PI) 3‐kinase (PI3K), and thereby activate p85 subunits of PI3K. In turn, p85/PI3K phosphorylates PI‐4.5‐(PO_4_)_2_ in the plasma membrane (PM) to produce PI‐3,4,5‐(PO_4_)_3_ (PIP_3_), a highly acidic phospholipid that (a) binds to basic amino acids in isoforms of Akt (1/2/3),^2^, ^3^ atypical protein kinase C (aPKC) (ζ/λ/ι),^4^, ^5^, ^6^ and phosphoinositide‐dependent kinases‐1 (PDK‐1)^7^ and (b) triggers phosphorylation of key activating serine and threonine residues in all isoforms of both Akt and aPKC by PDK1 and PDK‐2, and, for aPKCs, by auto(trans)phosphorylation.^8^

**FIGURE 1:**
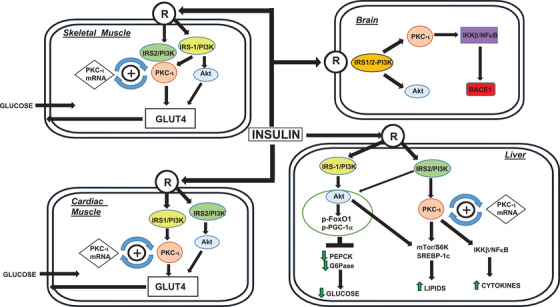
PKC‐i in primates and its homologue, PKC‐l, in mice are the major 70 kDa aPKCs in liver, skeletal muscle, cardiac muscle, and brain and are activated by IRS‐1/PI3K in skeletal and cardiac muscle, but by IRS‐2/PI3K in liver (brain data on IRS are lacking). In muscle and liver, Akt is mainly activated by IRS‐1/PI3K, but, in heart, Akt is mainly and constitutively activated by IRS‐2/PI3K. In liver, Akt selectively phosphorylates FoxO1 and PGC‐1α on the WD40/ProF platform, shown by the shaded area, and, whereas both Akt and aPKC are used for insulin‐stimulated lipogenesis, Akt alone mediates insulin‐suppression of gluconeogenesis. Indeed, aPKC excess impairs the effect of Akt on FoxO1, PGC‐1a, and gluconeogenesis by displacing Akt from the WD40/ProF platform

Subsequently, in skeletal muscle and adipocytes, both Akt and aPKC operate downstream of IRS‐1^9^ to mediate insulin effects on glucose transport. In cardiac muscle, whereas IRS‐1 controls aPKC and thus glucose transport during insulin action, IRS‐2 and Akt appear to be constitutively active. In liver, whereas Akt is largely controlled by IRS‐1, aPKC is controlled by IRS‐2^10^; and, during insulin action, whereas both Akt and aPKC increase lipogenesis, Akt alone increases glucose storage in glycogen and suppresses glucose release and gluconeogenesis (Figure 1).

## 2 GENERAL ASPECTS OF INSULIN RESISTANCE

### 2.1 Mechanisms for initiating insulin resistance and hyperinsulinemia

Insulin resistance and hyperinsulinemia occur in response to impairments of activation of the IR and/or post‐IR intracellular signaling factors, increases in hepatic glucose production, and decreases in muscle glucose utilization. Insulin normally suppresses hepatic gluconeogenic enzyme expression and stimulates muscle glucose uptake (Figure 1), and both actions are impaired in diet‐induced obesity, the metabolic syndrome, type 2 diabetes (DIO/MetS/T2DM). Additionally, acute hepatic actions of insulin that promote glucose storage and diminish glucose release are opposed by glucagon, corticosteroids, and catecholamines. Whereas there are many starting points for developing insulin resistance and hyperinsulinemia, a particularly important one in Western societies is dietary excesses. Indeed, DIO and MetS features of hyperlipidemia, hepatosteatosis, hypertension, and glucose intolerance, or overt T2DM, are readily produced in experimental mouse and monkey models by hypercaloric diets, whether excessive in fats or carbohydrates.

### 2.2 Prevalence and sequelae of insulin resistance and hyperinsulinemia

Obesity, MetS, and T2DM have reached pandemic levels as nations have adopted calorie‐rich “Western” diets. In USA, DIO/MetS afflicts one in four adolescents and one in three adults, progresses to T2DM in one of four adults and underlies much of our cardiovascular disease. The American Diabetes Association estimates that DIO/MetS/T2DM will soon involve 40% of our population, and much of our medical expenditures are needed to treat DIO/MetS/T2DM and cardiovascular sequelae, coronary artery disease, and stroke; hence coining of the term, “cardiometabolic syndrome.”

Further, at the Mayo Clinic, T2DM or “fasting glucose intolerance” is present in 80% of Alzheimer patients,^11^ and other reports suggest that T2DM prevalence in Alzheimer's disease (AD), and AD prevalence in T2DM, is increased approximately twofold.^12^, ^13^, ^14^ And, as discussed below, in mice and monkeys with DIO/MetS/T2DM, we found that hyperinsulinemia provokes increases in activity of β‐site amyloid precursor protein (β‐APP) cleaving enzyme‐1 (BACE1), which, in brain, increases Aβ‐peptide production and may abet development of Aβ‐plaques and phospho‐tau “tangles”^15^ in AD, which alarmingly afflicts one of five women and one in 10 men over age 65, and one of two humans over age 85. By analogy, this may constitute a “neurometabolic syndrome.”

There are other clinical problems that may be abetted by hyperinsulinemia *per se* in insulin‐resistant states, for example, the polycystic ovary syndrome, which reflects thecal hyperplasia and androgen overproduction by the ovary, and enhanced growth of various neoplasias, perhaps reflecting growth‐promoting properties of insulin.

Finally, note that insulin resistance can involve and eventually lead to deficiencies and demise of insulin‐producing β‐cells in pancreatic islets, and, vice versa, deficiencies of insulin secretion can produce extra‐pancreatic insulin resistance.

In any case, we need to develop more effective means to reverse mechanisms that initiate or sustain DIO/MetS/T2DM, and this will require a more comprehensive understanding of the pathophysiology of insulin resistance and development of agents that restore normal cellular physiology.

### 2.3 The hyperinsulinemia component of insulin resistance syndromes

A theme that will emerge in this review is that hyperinsulinemia underlies many, albeit not all, of the clinical features of the insulin‐resistance syndrome. Heretofore, we commonly assumed that clinical manifestations and adverse sequelae of DIO/MetS/T2DM arose solely/largely from impaired insulin action, and, indeed, IR downregulation is commonly present in many/most cell types in DIO/MetS/T2DM. However, in many cell types, compensatory hyperinsulinemia in insulin‐resistant states can bypass partial IR defects by activating “spare IRs,” that is, IRs present in excess of those needed to fully/strongly activate post‐IR processes.^16^ On the other hand, insulin resistance that cannot be reversed by hyperinsulinemia is often produced by diminished activity of IRS‐1 and its activation of phosphatidylinositol (PI) 3‐kinase (PI3K); this post‐IR defect is provoked by several mechanisms in DIO/MetS/T2DM. Most notably, IRS‐1/PI3K is conspicuously impaired in skeletal muscle in early phases of DIO in the mouse,^17^ and undoubtedly contributes importantly to the hyperinsulinemia that can unfortunately bypass partial impairments in IR action in liver and adipose tissue, and thus promote fat production and storage. Further note, unlike IRS‐1/PI3K, activation of IRS‐2 and IRS‐2/PI3K in both muscle and liver is resistant to downregulation in DIO/MetS/T2DM^17^, ^18^, ^19^, ^20^ (Figure 1) and is responsive to hyperinsulinemia.^18^, ^19^, ^20^, ^21^ Further note that the insulin‐sensitive shc/ras/raf/MEK/ERK pathway, which is important for cell growth, is also resistant to downregulation.

### 2.4 Present‐day therapy of insulin‐resistant hyperinsulinemic states

Presently, treatment of T2DM is indirect and piecemeal, with use of metformin, insulin secretagogues and glucosurics to diminish hyperglycemia; and various agents to treat MetS features of hyperlipidemia and hypertension. Agents that directly increase insulin signaling at IR or post‐IR levels, “insulin sensitizers,” or act like, but independently of, insulin intracellularly to reverse T2DM‐induced biochemical aberrations, “insulin mimickers,” would seem more logical, as insulin resistance is widespread, if not ubiquitous, in DIO/MetS/T2DM.

In short, we need treatments that reverse the hyperinsulinemia resulting from impairments in insulin signaling or glucose metabolism, as hyperinsulinemia can hyperactivate uninhibited or partially inhibited insulin‐signaling pathways and provoke adverse effects. As discussed, only a fraction of IRs is needed to provoke maximal insulin effects in many cell types, most notably, hepatocytes, where lipids are produced, and adipocytes, where lipids are stored, and, when excessive, can provoke adverse effects locally and distally, for example, in liver.^22^

In the above scenario, hyperinsulinemia can indeed provoke adverse responses, including increases in hepatic lipogenesis that contribute to hypertriglyceridemia, hypercholesterolemia, hepatosteatosis, and extrahepatic fat deposition, that is, obesity; increases in renal release of renin and resultant angiotensin‐mediated vasoconstriction and aldosterone‐mediated sodium retention; increases in tumor/cancer growth; and increases in neuronal BACE1, which abets Alzheimer development.

Unfortunately, we presently lack true hepatic insulin sensitizers and mimickers in our therapeutic armamentarium. Metformin is considered as a hepatic insulin sensitizer/mimicker, but diminishes hepatic gluconeogenesis by directly inhibiting key mitochondrial enzymes^23^ or respiratory factors needed to generate ATP required for gluconeogenesis,^24^ rather than by correcting T2DM‐induced increases in gluconeogenic enzymes; in fact, metformin may self‐limit its effectiveness thereupon by activating hepatic aPKC.^25^, ^26^

On the other hand, both metformin^27^ and thiazolidinediones^28^ apparently act as insulin sensitizers or mimickers in muscle where they were found to increase insulin‐stimulated glucose disposal in euglycemic‐hyperinsulinemic clamp studies while increasing aPKC activity in muscles of T2DM humans; in this regard, note that (a) metformin itself activates aPKC and increases glucose transport in isolated myocytes^29^ and (b) thiazolidinediones diminish hepatic lipogenesis,^30^ which can secondarily improve insulin signaling in muscle^17^, ^20^, ^21^ (and see below). Although thiazolidinedione usage has declined because of edema development and cardiac concerns, metformin continues as the mainstay for treating obesity‐related T2DM.

As to other antidiabetic agents, glucagon receptor peptide‐1 (GLP‐1) receptor agonists and dipeptidyl peptidase‐4 inhibitors, which increase endogenous GLP‐1, apparently improve hyperglycemia by acting in pancreas to diminish glucagon secretion and improve glucose‐stimulated insulin secretion. Their efficacy in part may reflect β−cell proliferative effects of GLP‐1 that are mediated by activation of aPKC,^31^ and, indeed, PKC‐λ/ι is required for maintenance of mouse β‐cell integrity.^32^ However, these agents may additionally improve insulin resistance via weight loss owing to effects on the gastrointestinal function and appetite suppression.^33^ Also, in DIO/MetS/T2DM, we presently do not know if β‐cell aPKC activity is altered downward (as in muscle—see below), or upward (as in liver), in which case, it may participate in mediating compensatory increases in insulin secretion.

Different from current approaches, we identified excessive activity of hepatic aPKC as a key factor for producing diabetogenic aberrations in hepatic enzymes and resultant clinical manifestations of insulin‐resistant DIO/MetS/T2DM mice. Moreover, we developed agents that selectively inhibit hepatic aPKC, without inhibiting muscle or adipose aPKC, which, along with Akt, mediates insulin effects on glucose transport/uptake. In fact, these inhibitors of hepatic aPKC improve impairments in insulin signaling to IRS‐1/PI3K, Akt, and aPKC in skeletal muscles of DIO/MetS/T2DM mice. As discussed, these inhibitors correct molecular aberrations in hepatic insulin signaling at IR and post‐IR levels; in short, they act as insulin “sensitizers” and “mimickers” and restore normal insulin signaling in liver, muscle, and adipose tissues.

### 2.5 Requirements for aPKC in insulin‐stimulated glucose transport in muscle and adipose tissues

Before discussing the role of hyperactive hepatic aPKC in insulin‐resistant/hyperinsulinemic states, note that, in prior studies, we reported that aPKC activity is required for insulin‐stimulated glucose transport in skeletal muscle, cardiac muscle, and adipocytes; this was most definitively shown by muscle‐specific^34^ and adipocyte‐specific^35^ knockout of PKC‐λ, the major aPKC in mice. These findings were additionally confirmed in studies of iRNA‐mediated knockdown of PKC‐λ in mouse‐derived 3T3/L1 adipocytes and knockdown of PKC‐ζ (the major rat aPKC) in rat‐derived L6‐myocytes^36^; moreover, knockdown‐induced losses of insulin‐stimulated glucose transport were rescued by adenoviral‐mediated expression of the opposite aPKC, thus indicating functional interchangeability of aPKCs for this process.

It was further found that the reduced ability of insulin to increase glucose transport in skeletal muscle is associated with impaired activation of aPKC, as well as IRS‐1/PI3K and Akt, in muscles of high‐fat‐fed (HFF) and ob/ob mice.^17^, ^20^, ^21^ Most importantly, insulin stimulation of muscle‐dependent glucose disposal in hyperinsulinemic‐euglycemic clamp studies was found to be impaired in T2DM humans^37^, ^38^ and monkeys,^39^ and, moreover, accompanied by deficiencies in both activity and *amount* of muscle PKC‐ι, the primate‐specific aPKC isoform (98% amino acid homology to nonprimate PKC‐λ).

In contrast, levels of muscle PKC‐λ and PKC‐ζ are not reduced in T2DM rodents,^18^, ^20^, ^21^, ^40^, ^41^ most likely reflecting differences in promoter/transcriptional regulation of subprimate aPKCs *vis‐a‐vis* primate‐specific PKC‐ι, expression of which is positively regulated by its own activity.^18^, ^19^ Moreover, this regulation of transcription by positive‐feedback explains: diminished PKC‐ι levels that follow diminished activity of IRS‐1/PI3K and PKC‐ι in muscles of T2DM humans^37^, ^38^; and elevated PKC‐ι levels in livers of T2DM humans.^18^, ^19^

## 3 MOLECULAR MECHANISMS UNDERLYING INSULIN RESISTANCE

### 3.1 Hepatic aPKC and its critical role in diet‐dependent insulin resistance and hyperinsulinemia

With findings of aPKC deficiencies in muscle studies, it was particularly surprising to find increases in PKC‐λ activity in livers of DIO/MetS/T2DM mice^17^, ^20^, ^21^, ^40^, ^41^, ^42^, ^43^, ^44^; and increases in activity and mRNA and protein levels of PKC‐ι, and operation of a feed‐forward, positive‐feedback, autocatalytic, mechanism, in livers of obese and T2DM humans.^18^, ^19^ Moreover, increases in hepatic aPKC activity in DIO/MetS/T2DM mice apparently serve importantly in the development of systemic insulin resistance, hyperinsulinemia, and clinical MetS abnormalities, that is, abdominal obesity, hepatosteatosis, and elevations of serum triglycerides and cholesterol; indeed, these abnormalities are abrogated or markedly improved by (a) treatment of DIO/MetS/T2DM mice with inhibitors of hepatic aPKC, as produced either by chemical agents^21^, ^22^, ^43^, ^44^ or by hepatic expression of kinase‐inactive aPKC^17^, ^40^, ^41^; or (b) deficiency of hepatic PKC‐λ induced by various knockout methods.^40^, ^41^, ^42^

**FIGURE 2:**
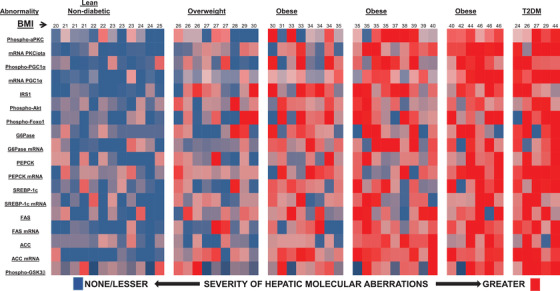
Heat map of obesity (as per BMI) and T2DM‐dependent abnormalities in insulin signaling to phospho‐aPKC, phospho‐Akt, phospho‐FoxO1, and phospho‐PGC‐1α, and protein and/or mRNA levels of PKC‐i, IRS‐1, PGC‐1α, gluconeogenic enzymes (PEPCK/G6Pase), and lipogenic enzymes (SREBP‐1c, ACC, FAS) in human liver. (Data from Ref. ^8^)

To summarize, it seems clear that excessive activity of hepatic aPKC plays a key role in promoting systemic insulin resistance in HFF^17^, ^20^, ^40^, ^41^, ^42^, ^43^ and ob/ob^21^, ^40^, ^41^ mice and in producing a “T2DM enzymatic phenotype” in livers of these mice and livers of obese and T2DM humans,^18^, ^19^, ^43^ wherein insulin signaling is deficient to Akt and excessive to aPKC, and, moreover, is accompanied by increases in expression of gluconeogenic, lipogenic, and proinflammatory enzymes. This T2DM phenotype is portrayed in a heatmap (Figure 2) showing alterations in livers harvested from increasingly obese and T2DM humans.^18^, ^19^, ^43^ In keeping with these findings, increased hepatic gluconeogenesis occurs early in the course of development of insulin resistance in obese adolescent humans.^45^

Furthermore, it seems clear that dietary excesses in either lipids or carbohydrates can trigger or abet development of hepatic aberrations and insulin resistance, as shown by findings in HFF mice,^17^, ^20^, ^40^, ^41^, ^42^, ^43^ and high‐carbohydrate‐fed ob/ob mice^21^, ^40^, ^41^ and monkeys.^39^ The presence of similar hepatic aberrations and increases in ceramide in livers of obese and T2DM humans^18^, ^19^, ^43^ suggests similar involvement of dietary excess.

Finally, note that liver plays an important role in DIO/MetS/T2DM, even when systemic insulin resistance is initiated or abetted by (a) adipocyte aberrations in obesity that lead to excesses in circulating ceramide levels^22^ and (b) muscle aberrations, for example, in Het‐MλKO mice, where an initial defect in muscle glucose transport^34^ leads to hyperinsulinemia and secondary increases in activity of hepatic aPKC,^44^ which is controlled by IRS‐2/PI3K and, unlike IRS‐1/PI3Kdependent Akt, is conserved in T2DM.^17^, ^18^ Accordingly, even when insulin resistance starts elsewhere, inhibition of hepatic aPKC can reverse increases in hepatic gluconeogenic, lipogenic, and proinflammatory enzymes, and thereby correct/improve clinical problems of abdominal obesity, other MetS features, and glucose intolerance.

### 3.2 Diet‐dependent dysregulation of hepatic gluconeogenesis and lipogenesis in insulin‐resistant states

**FIGURE 3:**
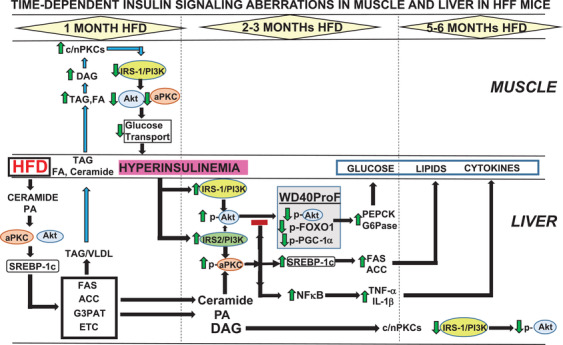
Time‐dependent development of aPKC‐dependent aberrations in postreceptor insulin signaling and appearance of alterations of gluconeogenic, lipogenic, and proinflammatory factors, in the mouse model of diet‐dependent, HFD‐induced obesity, the metabolic syndrome, and T2DM. Note (a) in phase I, the prominent role of SREBP‐1c, as activated by aPKC and Akt, for promoting increases in the activation of conventional and novel PKCs in muscle that, perhaps along with ceramide, lead to postreceptor impairments in insulin signaling via IRS‐1/PI3K in muscle; and (b), in phase 2, the blockade (red bar) of Akt entry to the WD40/ProF scaffold (shaded area), the subsequent impairment of insulin/Akt‐dependent phosphorylation of FoxO1 and PGC‐1α, and the resultant impairment of insulin‐suppression of PEPCK/G6Pase expression and gluconeogenesis. Not shown is the timing of onset for aPKC‐dependent decreases in insulin receptor levels, which is still unknown, but is clearly present at 2–3 months of HFD

In early phases of DIO/MetS/T2DM in mice,^17^, ^20^, ^21^, ^40^, ^41^, ^42^, ^43^, ^44^ and late phases of DIO/MetS/T2DM in humans,^17^, ^18^, ^25^, ^43^ we similarly observed: (a) increases in levels of ceramide, a direct activator of aPKC; (b) increases in aPKC activity and aPKC association with scaffolding protein WD40/ProF (shaded areas, Figures 1 and 3); (c) decreased Akt2 association with scaffolding protein, WD40/ProF; (d) decreased Akt2‐dependent phosphorylation of FoxO1 and PGC‐1α which, in liver, apparently takes place on, or requires, the WD40/ProF platform; (e) increased PGC‐1α expression; (f) increased expression of FoxO1/PGC‐1α−dependent gluconeogenic enzymes, phosphoenolpyruvate carboxykinase (PEPCK), and glucose‐6‐phosphatase (G6Pase); (g) increased nuclear levels (activity) and expression of sterol receptor element‐binding protein‐1c (SREBP‐1c) and multiple SREBP‐1c‐dependent lipogenic enzymes, for example, fatty acyl synthase (FAS), acetyl CoA‐carboxylase (ACC); and (h) increased nuclear levels and activity of NFκB and subsequent increases in levels of multiple NFκB‐dependent proinflammatory cytokines, for example, tumor necrosis factor‐α (TNF‐α) and interleukin‐1β ( IL‐1β). In later phases of DIO/MetS/T2DM, hepatic IRS‐1 activity and levels diminish, thus causing decreases in IRS‐1/PI3K and Akt activation, and decreases in phosphorylation of other Akt substrates, for example, glycogen synthase kinase‐3β (GSK3β). This later phase of the “obesity/T2DM hepatic phenotype” is depicted in a heat map (Figure 2) that graphically portrays body‐mass‐index (BMI)‐correlated *abnormalities* in levels and/or activity of insulin‐signaling factors, IRS1, IRS‐2, Akt, and aPKC, and expression, as per mRNA and protein levels of gluconeogenic and lipogenic enzymes in livers of obese and T2DM humans (data from Refs. ^17^, ^18^, and ^43^).

Moreover, in HFF^17^, ^20^, ^40^, ^41^, ^42^, ^43^ and ob/ob^21^, ^40^, ^41^ mice, we also observed impairments in insulin signaling to IRS‐1/PI3K, Akt, and aPKC and insulin‐stimulated glucose transport in muscle^17^; and these impairments apparently reflected adverse liver‐to‐muscle cross talk, as all muscle‐signaling aberrations were ameliorated by (a) treatment of mice with liver‐selective inhibition of aPKC by both chemical agents and adenoviral‐mediated expression of kinase‐inactive (KI) aPKC and (b) liver‐selective knockout of PKC‐λ.^17^, ^20^, ^41^

Further, with the development of hyperinsulinemia and apparent activation of “spare” hepatic insulin receptors, we^17^, ^20^, ^21^, ^40^, ^41^, ^42^, ^43^ and others^46^, ^47^ found that certain insulin‐sensitive pathways in mouse liver are activated in early stages of high‐fat‐feeding, including, Akt. Indeed, early increases in Akt activity in HFF^17^, ^20^, ^40^, ^41^, ^42^, ^43^, ^46^, ^47^ and ob/ob^21^, ^40^, ^41^ mice are attended by increases in phosphorylation of mammalian target of rapamycin (mTOR),^20^, ^21^ which participates in SREBP‐1c activation and subsequent increases lipogenic enzymes (Figure 1). These increases in Akt‐dependent effects on SREBP‐1c and lipogenic enzyme expression accentuate those induced by increases in hepatic aPKC activity owing to increases in hepatic ceramide levels and hyperinsulinemia acting through IRS‐2/PI3K. Note that insulin activation of hepatic SREBP‐1c requires both Akt^43^, ^48^, ^49^ and aPKC.^17^, ^18^, ^19^, ^20^, ^21^, ^25^, ^40^, ^41^, ^42^, ^43^, ^44^, ^50^, ^51^, ^52^

### 3.3 The role of the WD40/ProF platform in dysregulation of hepatic gluconeogenic enzyme expression

As a key mechanism for developing diet‐dependent increases in hepatic gluconeogenic enzymes (PEPCK/G6Pase) in HFF and ob/ob mice, note that insulin, via PI3K, normally recruits Akt2 and aPKC to a scaffolding protein, WD40/Propeller‐FYVE (WD40/ProF), which binds PI3K products, PIP_3_, and/or PI‐3,4‐(PO_4_)_2_, and localizes key signaling factors during insulin action in liver^19^, ^20^, ^21^, ^43^ and adipose tissue,^53^, ^54^, ^55^ but not in myocytes.^53^, ^54^, ^55^ And, in liver, Akt selectively phosphorylates both FoxO1 and PGC‐1α, but not other Akt substrates, for example, mTOR and GSK3β, on/near this platform^19^, ^20^, ^21^, ^43^ and thereby reduces active nuclear levels of FoxO1 and PGC‐1α and their contribution to gluconeogenic enzyme expression.^56^, ^57^ Moreover, in HFF and ob/ob mice and livers of T2DM humans, there are diet‐dependent increases in ceramide^19^, ^20^, ^21^ and phosphatidic acid (PA) that directly activate hepatic aPKC, which displaces Akt2 from the WD40/ProF platform,^19^, ^20^, ^21^, ^43^ thus impairing FoxO1 and PGC‐1α phosphorylation and thereby increasing their nuclear localization, transcriptional activities, and expression of PGC‐1α and gluconeogenic enzymes, PEPCK, and G6Pase.

As discussed, in liver, Akt displacement from WD40/ProF occurs relatively early with dietary excesses, even when Akt activity and phosphorylation of mTOR and GSK3β are normal or increased by hyperinsulinemia in HFF^20^, ^43^ and ob/ob^21^ mice. Furthermore, Akt displacement from WD40/ProF is even more pronounced in livers of humans^19^ with well‐established obesity or T2DM, in conjunction with decreases in IRS‐1 levels and activities of IRS‐1/PI3K and Akt.^18^, ^19^, ^20^, ^43^ And, even in normal conditions, aPKC tonically restrains Akt actions on FoxO1 and PGC‐1α, as simple inhibition or deficiency of hepatic aPKC increases basal/resting FoxO1 phosphorylation and diminishes gluconeogenic enzyme expression,^18^, ^19^, ^20^, ^21^, ^41^, ^42^, ^43^, ^44^ even without concomitant insulin treatment. Finally note that hepatic FoxO1 phosphorylation is constitutive when PKC‐λ/ι is deficient, as in mice with heterozygous knockout of PKC‐λ that are fully protected from developing increases in gluconeogenic and lipogenic enzymes, and, for that matter, do not develop glucose intolerance or T2DM during high‐fat‐feeding, but, on the other hand, rapidly become overtly diabetic when PKC‐λ is overexpressed specifically in liver.^42^ The vulnerability of hepatic Akt‐dependent phosphorylation of FoxO1 and PGC‐1α in DIO/MetS/T2DM may reflect the hyperactivation of aPKC in PM areas^56^, ^57^ close to sites rich in WD40/ProF^53^, ^54^, ^55^ and PI3K‐dependent PIP_3_.

Interestingly, in concert with our findings, knockout of hepatic WD40/ProF (aka, WDFY2) diminishes insulin‐stimulated FoxO1 phosphorylation, increases gluconeogenic enzyme expression, and produces systemic insulin resistance; however, consequences of this knockout are more drastic than the functional changes in WD40/ProF induced by hyperactivity of hepatic aPKC in that total cellular activity of IRS‐1/PI3K‐dependent Akt2 is diminished in these knockout mice.^58^

### 3.4 Stages in development of diet‐dependent aberrations in hepatic insulin signaling in high‐fat‐fed mice

That diet‐induced increases in hepatic aPKC activity over 2–3 months (Figure 3) selectively impairs insulin‐stimulated recruitment of Akt to the WD40/ProF platform and phosphorylation/inhibition of FoxO1 and PGC‐1α, provides a unique mechanism for linking dietary excesses of fats in HFF mice, and carbohydrates in ob/ob mice and obese/T2DM monkeys, to increases in hepatic gluconeogenesis, and development of systemic insulin resistance and hyperinsulinemia. In turn, hyperinsulinemia increases hepatic activities not only of aPKC and Akt but also the activities of mTORC1 and SREBP‐1c, which are activated by both Akt^43^, ^48^, ^49^ and aPKC.^17^, ^18^, ^19^, ^20^, ^21^, ^25^, ^40^, ^41^, ^42^, ^43^, ^44^, ^50^, ^51^, ^52^ This may explain how hepatic production of lipids and glucose is simultaneously increased at 2–3 months of high‐fat‐feeding in the mouse,^20^, ^42^, ^43^ even though insulin normally has opposite effects on glucose and lipid production.

However, earlier, at 3–4 weeks of consuming a “Western” 40%‐fat‐kcal diet (Figure 3), increases in hepatic gluconeogenic enzymes, PEPCK/G6Pase, are *not* apparent,^17^ and hyperinsulinemia here apparently largely reflects other pathogenetic mechanisms, including impaired insulin action on IRS‐1/PI3K, Akt, aPKC, and glucose transport in muscle,^17^ and perhaps IR impairment (see below). Nevertheless, at 3–4 weeks of HFD, there are large feeding‐dependent increases in (a) hepatic SREBP‐1c activity and lipogenic enzymes (FAS/ACC), (b) hepatic NFκB activity and proinflammatory cytokines (TNF‐α/Il‐1β), and (c) serum triglycerides/triacylglycerols (TAGs).^17^, ^41^ Moreover, inhibition of hepatic aPKC corrects hepatic alterations in SREBP‐1c and NFκB, reduces circulating triglycerides, and simultaneously corrects defects in insulin signaling to IRS‐1/PI3K, Akt1/2 and aPKC in skeletal muscle.^17^, ^20^, ^21^, ^44^ This suggests that the liver adversely cross talks to muscle, perhaps via release of lipids [triglycerides, fatty acids (FAs), ceramides], and/or cytokines, and/or other factors, for example, exosomes rich in ceramide, which inhibits insulin action on IRS‐1/PI3K/Akt/aPKC in muscle.^56^, ^57^, ^58^ In this regard, note that intravenous lipid infusion over 5 h impairs insulin signaling to IRS‐1/PI3K, aPKC, and Akt1, but not IRS‐2/PI3K and Akt2, in rat skeletal muscle.^59^

Later, at 5–6 months of high‐fat feeding and beyond, in livers of rodents^17^, ^20^, ^43^ (Figure 3) and obese/T2DM humans,^18^, ^19^, ^43^ hepatic IRS‐1/PI3K fails and Akt activation diminishes; nevertheless, conserved activation of hepatic IRS‐2/PI3K and insulin‐stimulated aPKC activation may explain continued lipid and glucose overproduction, even when hepatic IRS‐1/PI3K/Akt activation is reduced. In this regard, hepatic mTOR phosphorylation remains elevated in DIO/MetS/T2DM humans even as Akt diminishes,^19^ suggesting that other factors, for example, aPKCs or other PKCs,^60^, ^61^ can activate p70/S6kinase, mTORC1, and SREBP‐1c.

Interestingly, the interplay between aPKC and the downregulation of Akt alluded to above has been mathematically analyzed and the switch‐off of IRS‐2/aPKC appears to be delayed in response to reductions in first phase and pulsatile insulin secretion in T2DM.^62^ However, precise modeling is difficult as aPKC is also activated by noninsulin factors and time‐related changes, as herein discussed.

It is of course entirely possible, if not likely, that much of our overfed population in the USA and other Westernized populations may have similar hepatic abnormalities, especially those present in phase 1, or phase 1 plus phase 2, of Figure 3, and the ensuing hyperinsulinemia may be particularly important in producing MetS features, and associated cardiometabolic, neurometabolic, and other risks.

### 3.5 Hepatic insulin receptor deficiency owing to aPKC‐dependent increases in β‐secretase (BACE1)

Until now, this review has focused on postreceptor events that lead to insulin resistance, most notably, increases in hepatic gluconeogenic and lipogenic enzymes. However, impairments of the hepatic insulin receptor may be equally important in producing systemic insulin resistance and hyperinsulinemia. Indeed, as discussed, at 3–4 weeks of consuming a 40%‐fat‐kcal HFD, hyperinsulinemia is readily apparent,^17^ but unaccompanied by alterations in PEPCK/G6Pase; instead, muscle glucose transport is impaired by liver‐to‐muscle cross talk^17^ and undoubtedly contributes to development of hyperinsulinemia. In addition, we recently found that failure of the existing hyperinsulinemia in these HFF mice to increase conventional PKC‐α/β activity in liver, coupled with strong stimulatory effects of supplemental insulin thereupon, suggests a partial, but surpassable (via spare receptors), impairment in hepatic IRs; accordingly, as discussed below, it appears that IR abnormalities may result from HFD‐induced increases in hepatic aPKC activity at early stages of development of insulin resistance.

In any case, IR deficiencies at 1–3 months of HFD are partial and can be activated by high‐dose insulin to strongly/maximally activate (apparently via “spare” insulin receptors): (a) hepatic‐1/PI3K and IRS‐2/PI3K, and therefore, both Akt and aPKC in liver^20^; and (b) muscle IRS‐2/PI2K, but not IRS‐1/PI3K, and therefore neither Akt nor aPKC in muscle.^17^ And, in liver, elevated activities of hepatic Akt and aPKC in HFF mice are important for activation of SREBP‐1c and lipogenesis,^17^, ^20^, ^25^, ^41^, ^42^, ^43^ and aPKC activation therein reflects both diet‐dependent increases in ceramide and hyperinsulinemia‐induced activation of IRS‐2/PI3K and production of PIP_3_, which directly displaces the inhibitory pseudosubstrate, and simultaneously promotes threonine‐555/560‐phosphorylation, of aPKC‐λ/ι/ζ.^4^, ^6^, ^20^

As to how hepatic IRs may be downregulated, during insulin action, invaginated receptor‐containing vesicles are pinched off from the PM and transported to lysosomes or trans‐Golgi network (TGN), where proteolytic degradation occurs. And, in hyperinsulinemic states, this internalization process, and thus IR degradation, is enhanced, and this may lead to IR deficiency. In this scenario, IR downregulation results from hyperinsulinemia.

However, it was recently shown^63^ that IR deficiency is in fact produced by an increase in degradation, thus placing hyperinsulinemia downstream of IR downregulation. Moreover, it was reported that BACE1, a vesicle‐associated, transmembraneous, aspartyl‐peptidase (extensively studied in brain, where it acts upon vesicle‐associated, transmembraneous β‐APP to produce Aβ‐peptides that form pathological Alzheimer plaques) is abundant in liver, where it analogously acts upon and diminishes functional levels of the IR.^63^ Thus, just as BACE1 acts upon β‐APP in the TGN, BACE1 acts upon the ectodomain of the IR β‐subunit that projects into the lumen of TGN vesicles, thereby releasing the N‐terminal β−fragment and its sulfhydryl‐attached α‐subunit (“IRsol”) into the vesicle lumen. Lumen contents are ultimately emptied into the extracellular space after cycling of TGN vesicles back to the PM.^63^ Interestingly, plasma levels of this “soluble” fragment of the IR (“IRsol”) are increased in T2DM humans.^63^ Note that liver lacks β‐APP, precluding production of Aβ‐peptides.

Very importantly, hepatic BACE1 levels are increased in livers of HFF mice, db/db mice, and T2DM humans, and hepatic BACE1 levels correlate with IR degradation and decreases in cell‐surface/functional levels of IRs in liver, but not in muscle and adipose tissues that lack BACE1.^63^ Moreover, decreases in hepatic IRs are attended by diminished Akt activation.^63^ And, most important for this review, we found that aPKC is a major regulator of BACE1 mRNA and protein levels in mouse liver and brain; and, in conjunction with increases in hepatic aPKC activity in HFF mice, we found^64^ aPKC‐dependent increases in BACE1 levels and reciprocal decreases in IR β‐subunit levels; and both alterations are reversed by inhibition of hepatic aPKC. It will be interesting to see if aPKC‐dependent IR downregulation precedes, follows, or is contemporaneous with, aPKC‐dependent post‐IR aberrations.

Although these findings raise the possibility that BACE1 inhibitors, developed to treat Alzheimer's disease, could be used to treat T2DM, these inhibitors unfortunately enhanced impairment of cognitive/memory processes in Alzheimer patients. This enhancement may reflect that brain BACE1 levels rise during inhibitor usage,^65^ and increases in inhibited/inactive BACE1 may interfere with vesicle trafficking needed for learning and memory. Whether decreasing BACE1 levels to normal by diminishing BACE1 transcription during aPKC blockade is safer than chemical BACE1 inhibition, is uncertain. In this regard, knockout‐induced deficiency of brain BACE1 in transgenic Alzheimer mice reverses Aβ‐plaque formation and improves cognition/memory.^66^

## 4 INHIBITORS OF aPKC USED FOR TREATMENT OF INSULIN RESISTANCE IN EXPERIMENTAL FORMS OF DIO/MetS/T2DM

Several agents have been developed by high throughput screening of a chemical library against the crystallographic structure of PKC‐ι, most notably, 2‐**a**cetyl‐**c**yclo**p**entane‐1,3,‐**d**iketone (ACPD) and 1*H*‐**i**midazole‐4‐**c**arboxamide,5‐**a**mino][2,3‐dihydroxy‐4‐[(**p**hosphono‐oxy)methyl]‐cyclo**p**entane‐[1R‐(1a,2b,3b,4a)] (ICAPP) and its unphosphorylated inactive precursor ICAP, converted intracellularly to ICAPP by adenosine phosphorylase in liver^5^ and brain. These inhibitors, at low nanomolar levels, selectively inhibit recombinant aPKCs, and native aPKC in mouse liver and human hepatocytes, and, over 3‐months usage in mice, are well‐tolerated and without alterations in hepatic, renal, and hematologic parameters.^20^, ^21^, ^43^ These inhibitors do not inhibit recombinant conventional and novel PKCs in vitro, or Akt or AMPK in situ, and had no effect on a panel of 35 protein kinases, as independently tested by Life Technologies.^15^, ^20^, ^21^, ^44^ Also, in pharmacokinetic/pharmacodynamic/toxicology studies, ICAP was effective when given intravenously and orally, with liver uptake 3‐ to 10‐fold greater than brain uptake, and without high‐dose toxicity.^67^ Further note, ACPD and ICAPP/ICAP are liver‐selective; indeed, in liver‐effective doses, they improve, rather than inhibit, basal and insulin‐stimulated aPKC activity in muscle and adipose tissues of DIO/MetS/T2DM mice.^20^, ^21^, ^44^

## 5 SEQUELAE OF HYPERINSULINEMIA IN INSULIN‐RESISTANT STATES

### 5.1 Effects of insulin resistance and hyperinsulinemia in brain

As discussed, insulin‐resistant hyperinsulinemic states are prevalent in AD,^11^, ^12^, ^13^, ^14^ and it has been speculated that insulin action in brain is impaired in AD *and* DIO/MetS/T2DM states that precede and presumably increase Alzheimer risk. This speculation assumes that all tissues, including brain, are insulin‐resistant and therefore hypoinsulinized in insulin‐resistant states.

This assumption, however, proved to be too narrow. Thus, to test the hypothesis that excessive activity of hepatic aPKC leads to systemic insulin resistance and extrahepatic aberrations, we initially found that various aPKC inhibitors, ACPD, ICAP, and aurothiomalate (ATM), can reverse/improve all hepatic aberrations and clinical DIO/MetS/T2DM abnormalities, *i*ncluding hyperinsulinemia.^20^, ^21^, ^44^ Later, we surprisingly found in brains harvested from these same HFF, ob/ob, and Het‐MλKO mice and also in brains of obese/T2DM monkeys, that, contrary to the speculation that the brain is insulin‐resistant and hypoinsulinized in states of systemic insulin resistance, the activity of the brain IR and activities of both Akt and PKC‐λ/ι are all maximally activated, by basal resting hyperinsulinemia.^15^, ^68^ Indeed, elevated activities of brain Akt and aPKC in DIO/MetS/T2DM mice returned to normal following reversal of hyperinsulinemia, as elicited by correction of hepatic signaling aberrations and clinical abnormalities with *liver‐specific* aPKC inhibitor, ATM, which did not cross the blood brain barrier.^15^, ^44^ We also found that insulin activates BACE1 and increases Aβ‐peptide production in isolated neurons and mouse brain slices by an aPKC‐dependent mechanism.^15^, ^68^

Thus, contrary to then‐prevailing ideas, we found that the brain is hyperinsulinized in both short‐term mouse and long‐term monkey forms of DIO/MetS/T2DM. These findings do not rule out the possibility that the brain may become hypoinsulinized in subjects that have IR deficiencies but lack sufficient elevations of blood insulin to bypass receptor defects via spare receptors, for example, as insulin secretion declines in later phases of T2DM.

In this regard, brain IR activity and responsiveness to low‐physiological insulin concentrations are diminished in brains of humans with nondiabetic AD, but this IR defect is bypassed by higher insulin concentrations^69^ acting via spare IRs and/or insulin‐like growth factor‐1 receptors. Obviously, this IR defect may curtail beneficial (most likely, Akt‐mediated) effects of insulin in brains of humans with normo‐insulinemic, nondiabetic AD.

Of further note, activities of aPKC and Akt are strongly/maximally activated by uncertain, presumably noninsulin, factors in brains of humans with nondiabetic AD,^69^ and these aPKC elevations may increase BACE1 and thereby decrease IRs while simultaneously increasing Aβ‐plaque formation and AD risk. Indeed, hyperinsulinemia in DIO/MetS/T2DM mice and monkeys increases both BACE1 activity^68^ and recently we found BACE1 levels which increases plaque‐forming Aβ‐peptides and tangle‐forming phospho‐tau.^15^, ^68^ In short, elevations in brain aPKC activity may abet development of pathological processes in both nondiabetic and DIO/MetS/T2DM‐assocated AD.

### 5.2 Effects of insulin‐resistant hyperinsulinemic states on the heart

The development of cardiovascular problems is abetted by MetS features: (a) hypertension, owing to hyperinsulinemia‐stimulated renal renin release^70^ and increases in angiotensin‐induced vasoconstriction and aldosterone‐induced sodium retention; and (b) hyperlipidemias, owing to hyperinsulinemia‐stimulated increases in hepatic lipid production. Additionally, there is strong evidence^17^, ^20^, ^21^, ^43^ of adverse liver‐to‐skeletal‐muscle cross talk that may reflect hepatic overproduction and release of lipids (triglycerides, ceramides, fatty acids), proinflammatory cytokines, and/or ceramide‐rich exosomes into the circulation, followed by activation of diacylglycerol(DAG)‐activated PKCs and impairments of IRS‐1 and IRS‐1/PI3K‐dependent activation of Akt and aPKC in muscle. Indeed, inhibition of hepatic aPKC by liver‐selective agents is accompanied by improvements in insulin‐stimulated activation of IRS‐1/PI3K, Akt, and aPKC in skeletal muscle in various mouse models of DIO/MetS/T2DM.^17^, ^20^, ^21^ Moreover, recent findings suggest that similar adverse cross talk exists between liver and cardiac muscles and is corrected by inhibitors of hepatic aPKC; this improvement in IRS‐1/PI3K and aPKC activation presumably improves not only glucose transport but also PGC‐1α expression and mitochondrial oxidation and ATP production in the myocardium.

## 6 MECHANISMS FOR ACTIVATION OF aPKC

Finally note, in addition to aPKC activation by PI3K‐dependent increases in PIP_3_,^4^, ^5^, ^6^ during insulin and other polypeptide action, and by diet‐induced increases in hepatic ceramides and PA, aPKCs are activated by PA produced by (a) de novo synthesis from glycerol‐3‐PO_4_ and fatty acyl‐CoA; (b) phospholipase D, during ERK activation by hyperglycemia,^71^ metformin/AICAR,^25^, ^26^, ^72^, ^73^ and sorbitol/oxidation^74^; and (c) diglyceride‐kinase‐dependent phosphorylation of DAG produced by phospholipase C, as activated by H_2_O_2_,^75^ lipopolysaccharides,^76^ and related proinflammatory agents. Also, aPKC is activated by proinflammatory cytokines, TNF‐α and IL‐1β,^77^ that is, products of NFκB, which interestingly is activated by aPKC^17^, ^18^, ^41^, ^42^, ^44^ and may thus constitute a vicious cycle.

## 7 CLOSING COMMENTS

At this point, it seems clear that, in DIO/MetS/T2DM, hepatic aPKC hyperactivity plays a key role in producing aberrations in hepatic signaling that lead to increases in expression of gluconeogenic, lipogenic, and proinflammatory enzymes/cytokines, and subsequent development of systemic insulin resistance, glucose intolerance, and hyperinsulinemia, which in turn causes or abets development of liver‐dependent MetS features, and cardiovascular and neurological sequelae. We know that this sequence can be controlled by limiting hepatic aPKC. The next challenge is to develop safe therapeutic approaches that limit hepatic aPKC.

## CONFLICTS OF INTEREST

University of South Florida and the Department of Veteran Affairs have, or have applied for, patents on the use of inhibitors of atypical protein kinase C for treatment of Alzheimer's disease, obesity and diabetes.

## AUTHOR CONTRIBUTIONS

RV Farese wrote the paper, and all authors provided content and thought to the paper.

## ETHICS APPROVAL

Not applicable.

## Data Availability

All data generated or analyzed during this study are included in this published article.
